# Supporting the Learning of Visual Perception Skills in Cytology Through a Computational Tool: Development and Evaluation Study

**DOI:** 10.2196/79508

**Published:** 2026-09-10

**Authors:** Breno Nunes de Sena Keller, Mariana T Rezende, Renata RR Oliveira, Cristina RP Costa, Claudia M Carneiro, Andrea G Campos

**Affiliations:** 1 Programa de Pós-Graduação em Ciência da Computação, Universidade Federal de Ouro Preto, R. Diogo de Vasconcelos, 122, Ouro Preto, 35400-000, Brazil, 55 31 3559 1692; 2 Departamento de Computação, Universidade Federal de Ouro Preto, Ouro Preto, Brazil; 3 Departamento de Análises Clinicas, Universidade Federal de Ouro Preto, Ouro Preto, Brazil

**Keywords:** cytology, computational tool, visual perception learning, training, education

## Abstract

**Background:**

Advances in hardware and software have transformed how users perform activities across various domains by integrating technology in ways that enhance task execution. In education, these processes facilitate pedagogical innovation and learning through diverse interaction models, such as web systems, augmented or virtual reality, and mobile devices. An example of these approaches occurs in the context of cytopathology. Digitization and slide visualization technologies allow the use of real images in a computational environment, enabling an alternative model of interaction between the professional and the samples.

**Objective:**

This study aims to propose a computational framework to support the teaching-learning process of visual perception skills, using cervical cytology as a case study.

**Methods:**

The framework described in the study provides interactive, adaptive exercises that simulate practical laboratory experiences, enabling students to engage with specific diagnoses and rare scenarios. A proof-of-concept system was implemented and evaluated with undergraduate students and professionals in cervical cytology.

**Results:**

Test participants answered an average of 88.36% (SD 10.30%) of the activities and correctly responded to an average of 69.86% (SD 14.78%) of the questions. Compared with previous approaches, these results indicated no significant difference in student performance compared with this work approach, suggesting that the proposed framework offers similar, if not improved, performance, while providing better usability and overall user experience. Additionally, the results demonstrate the system’s effectiveness in supporting cervical cytology learning while minimizing its impact on the user’s routine. Furthermore, the test participants reported a positive impression of the system and its approach to supporting the learning process in cervical cytology.

**Conclusions:**

The study highlights the framework’s potential for scalability and adaptation across other disciplines that require visual analysis, offering a promising avenue for enhancing education through technology.

## Introduction

Computational tools can potentially enhance teaching and learning processes by offering features and resources that adapt real-world interaction models into virtual environments [1]. This adaptation permits building a learning process aligned with the concept of Education 4.0, where computational tools are used to enhance and improve learning [2]. In addition, using these resources also allows customizing the learning process to the characteristics of the users [1,3]. In this context, the integration of machine learning tools offers the advantage of being able to adapt the process to the student through data-driven insights, as they allow the process to be adjusted to the characteristics and limitations of each user, resulting in users achieving their goals more efficiently [4,5].

In the areas of pathology and cytopathology, the incorporation of technological tools occurs in different ways and at diverse levels of education and professional training [6-9]. These implementations generally use digital slide capture technologies and optical microscopes to generate whole slide images. These images allow different interaction models to be explored, especially in a learning context [9,10]. Another benefit of this digitization process is that whole slide images facilitate physical separation between professionals and samples, ensuring that interaction with the material is not geographically limited. However, this type of approach requires expensive equipment to generate images, internet access, and high-quality devices to ensure the quality of interaction with the professionals. Another approach using these images is the development of computational systems and algorithms to support or automate analysis and manipulation processes.

An interesting use case for digital health tools is to support screening in cytological analyses by building the necessary professional knowledge. In routine practice, cytologists (professionals) need to visually evaluate cytological specimens, which often exhibit significant morphological variability, and, through this analysis, identify different elements within the smear [11]. The variability of the material and the experience required to perform these evaluations make this analysis a complex task. The integration of technological resources into this teaching process augments the training and acquisition of specialized knowledge for cytology students, as they are empowered to accumulate experience through available tools as a complement or alternative to real-world activity, something especially important when dealing with rare and unusual cases in the professional’s routine.

This study introduces an innovative framework, named CRIC-Edu, designed to support the learning process of cytology students through interactive and adaptive exercises, with a focus on visual perception skills, in the context of higher education. The proposed system aims to refine students’ visual perception (specifically pattern discrimination ability) through case-based learning. Additionally, this study evaluates the proposed framework and compares the current implementation with a previously developed app, incorporating lessons learned from the earlier version to optimize user engagement and performance, thus refining the overall learning experience.

Our proposal advances the use of technological tools for training health care professionals, demonstrating the ability to overcome traditional limitations, such as the high cost of laboratory infrastructure and limited access to specialized training resources. This allows the system to democratize knowledge for students by decoupling the process from their physical and financial limitations. In addition, the interaction model enhances student engagement by replicating real-world scenarios in a scalable and adaptable format. Although the system was developed for a cytology case study and has shown its potential to simulate practical laboratory experiences for slide screening and diagnostics, its modular structure allows for scalability and application in other disciplines that require visual inspection or pattern recognition, expanding its impact beyond the specific domain of cytology. Furthermore, the modularized format of the system allows it to be modified and extended to incorporate new teaching support resources, such as multidisciplinary materials and customization features.

## Methods

### Case Study: Cervical Cytology Classes

Cervical cytology encompasses the study and investigation of specimens collected from the cervix. The fundamental principle of the cervical cancer screening test (Papanicolaou test) is the detection of preneoplastic lesions in desquamated epithelial cells, given that early-stage lesions can be detected in a healthy, asymptomatic population and are potentially curable. To achieve this goal, the cytologist needs specific training to perform the Papanicolaou test analysis. The knowledge necessary for this activity is built through specialized disciplines in higher education. In general, these courses introduce students to the epidemiological dimensions of cervical cancer and emphasize how screening for this neoplasia is essential to ensure women’s health outcomes. Thus, the course aims to develop students’ logical, critical, and analytical reasoning skills to conduct visual analysis correctly. Therefore, the course discusses and presents all stages of the exam, from specimen collection to the issuance of the diagnostic report, including staining techniques, slide assembly, cytological smear screening, interpretation, quality monitoring, and reporting.

**Figure 1.**
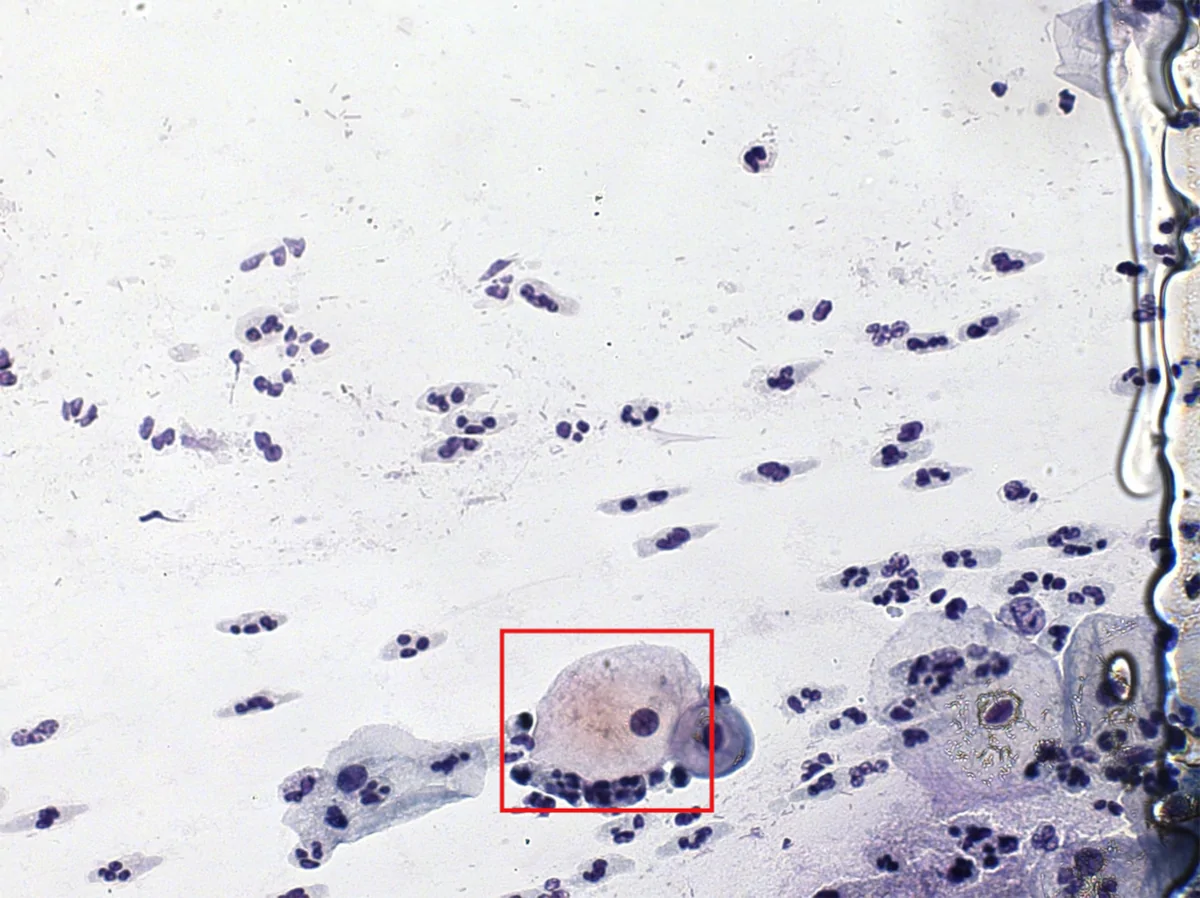
View of a Papanicolaou test from an optical microscope and the highlighted region of interest.

Traditionally, the course is organized into theoretical and practical classes. In theoretical classes, the professor presents the structure of tissues, their formation and maturation, and the pathological processes that can occur. The learning process occurs through exposure to various images that illustrate the different elements present in the cervicovaginal smear, such as cells (benign, malignant, or atypical), infectious agents, microbiota, inflammatory processes, cytological alterations, and other biological structures. In practical classes, students use optical microscopes to view slides with different smears, reinforcing their learning through practical experience and allowing them to apply their theoretical frameworks when exposed to diverse cases and situations. This combination of theory and practice aims to provide a comprehensive understanding and deepen knowledge about cellular morphologies and possible alterations, while simultaneously enabling the development of technical skills and preparing professionals for real-world situations and screening (diagnostic) exams. Figure 1 shows an example of the material visualized and the region of interest in a cytological smear analysis.

Education in cytology extends beyond undergraduate studies, as professional qualification in this field necessitates specialization in clinical cytology. Nonetheless, the foundational knowledge acquired during undergraduate training is continuously refined through daily professional activities. In addition, cytologists need to be up to date with the knowledge required to perform the analyses, whether by taking review courses or participating in events. These requirements render the study of cytology a perpetual activity essential for maintaining professional proficiency and diagnostic quality.

Regardless, operating a laboratory dedicated to practical classes imposes significant limitations, such as the need for specialized technicians to operate and maintain the equipment (optical microscopes) and the availability for use, since the same laboratory can be shared by different courses and classes in the same course [12]. In addition to the operational limitations, the high cost of acquiring and maintaining the necessary equipment and specialized personnel presents a further barrier. An alternative to mitigate these costs is to simulate laboratory interactions using computational resources and provide students with access to these tools, in parallel to the real-world environment, so that they have an alternative to train their skills. Also, access to the laboratory can be restricted to specific times and days, while the simulation, in turn, can be accessed at any time. Finally, the simulation model offers students an alternative to developing their visual inspection skills in scenarios where the laboratory is not available, such as the restrictions of the COVID-19 pandemic, since access to the system is not limited to the physical location [13].

### Visual Perception Learning

The quality of visual-based evaluations is directly linked to the interpretative capacity of those who perform them in analyzing the visual cues. Although well-established procedures and methods can reduce variability and increase the quality of the results, the main impact on the analysis process is usually the knowledge and experience of the individuals. This knowledge is constructed through visual perception, which describes the ability to interpret the information that the eyes perceive. This capacity is based on discrimination and visual memory, the ability to perceive differences or similarities in visual elements such as size, color, and shape, and the capacity to remember and compare visual stimuli. In addition, interpreting the meaning of these signals is linked to the professional’s experience and prior knowledge of the individual, characteristics that can be improved through training.

In cervical cytology, enhancing diagnostic quality involves cultivating skills in analyzing and identifying patterns within the cervical smear. In addition to theoretical knowledge, professionals must also refine their interpretation skills. The repetition of these tasks results in the improvement of the professional’s performance over time. This improvement results from the combination of accumulated experience in performing the task and the adoption of more efficient interpretative strategies [14,15].

This process is known as visual perception learning, which describes the enhancement of a person’s skills and abilities in performing a task through the accumulation of experience in performing a visual activity [14]. However, it is essential to emphasize that visual perception learning is not exclusive to specialized fields such as cytology or only to areas of professional activity, as it constitutes a fundamental component of learning throughout different life stages since childhood [16,17].

### Ethical Considerations

This study was submitted to and approved by the Universidade Federal de Ouro Preto ethics committee (CAAE 66468922.9.0000.5150). All participants were informed of the study’s purpose, methodological procedures, and the intended use of the collected data. All participants also provided written informed consent before participation. Participant anonymity was strictly protected, with all data anonymized. No study procedures posed risks beyond those ordinarily encountered in standard educational activities. No financial or other forms of compensation were provided to participants.

### The Proposed Framework

#### Preliminary Approach

In a study by Keller et al [18], the authors proposed and evaluated a computational solution with a similar objective to that presented in this study; however, their evaluation was performed in a different operational scenario during the COVID-19 pandemic. Their system was designed for use on smartphones and presented users with an extensive list of cytology-related activities. The evaluation encompassed 2 different test scenarios: the short academic period (SAP) and the regular academic period (RAP). Each of these scenarios was performed in parallel with the progress of the discipline during the COVID-19 pandemic. That study analyzed objective data about the scenario (such as activity accuracy) and provided insights into how students interact with mandatory activities, such as the evaluated system, on a regular schedule. It also highlighted how educational outcomes and content delivery can impact student behavior and study methodologies.

#### New System

##### Overview

This section describes CRIC-Edu and the overarching framework implemented by the system. This implementation is a proof of concept and, consequently, is limited in terms of content presentation available in the system, offering only two interaction models: (1) interactive images with delimited regions and (2) subject-specific slide presentations.

The architecture of the system consists of three main modules: (1) the content creation module, which is responsible for offering resources and tools that enable trained professionals to produce the necessary content for the platform, (2) the user interaction module, which handles the presentation of learning content to the user and collects their interaction with the content, and (3) the recommendation module, responsible for orchestrating content delivery.

##### Content Creation Module

In the content creation module, professionals develop platform-specific content based on their technical knowledge. This content can appear as a summary, an image, a presentation, or an activity. However, the content must be designed considering the limitations of the platform and the interaction models available between the platform and the user. Consequently, the system supports interactions that simulate or virtualize real-world activities. For instance, in the case of cervical cytology, the content can simulate the view users would experience through an optical microscope, simulating real-life tasks.

**Figure 2.**
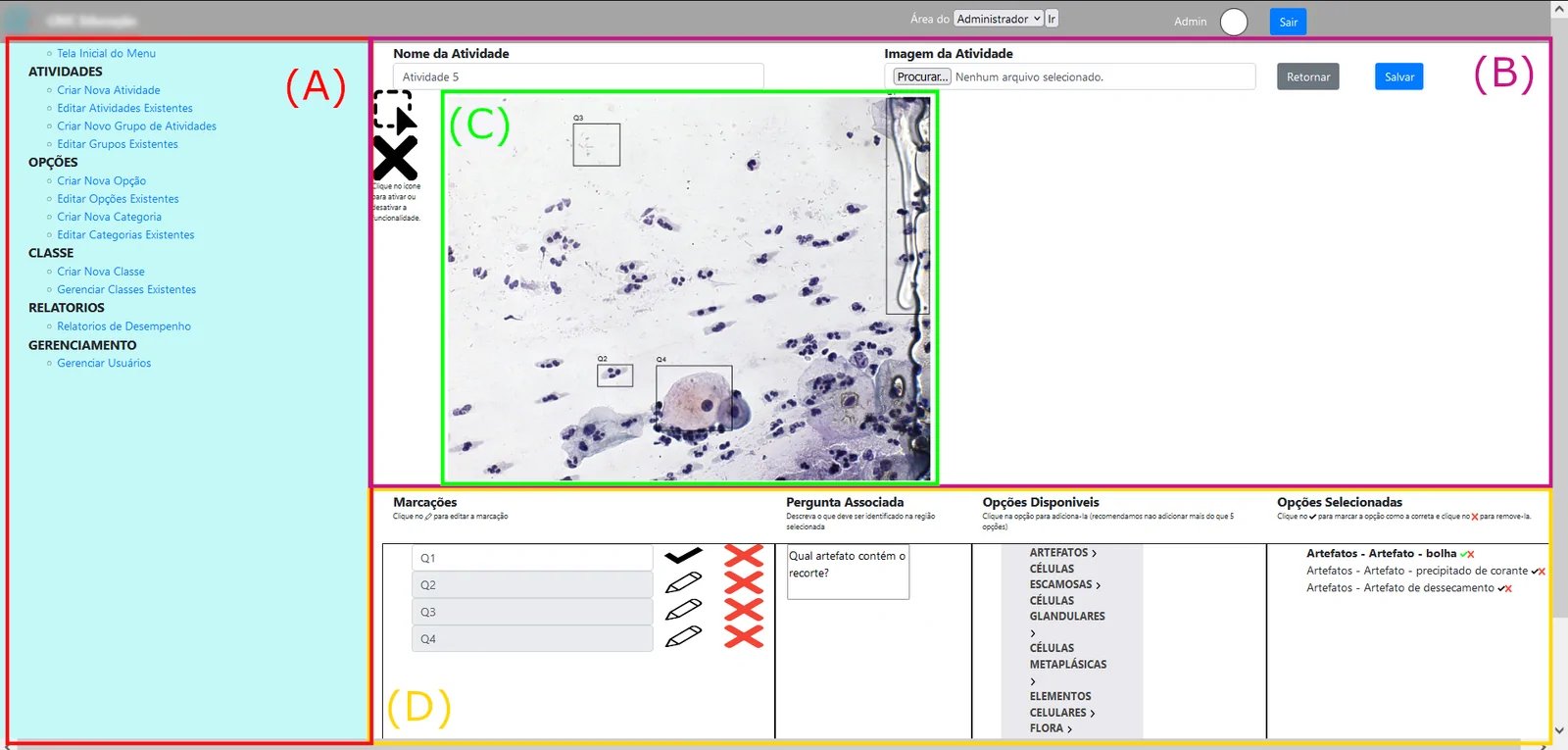
Example of the activity interface implemented in the system, showing how participants interact with the system to perform learning tasks.

**Figure 3.**
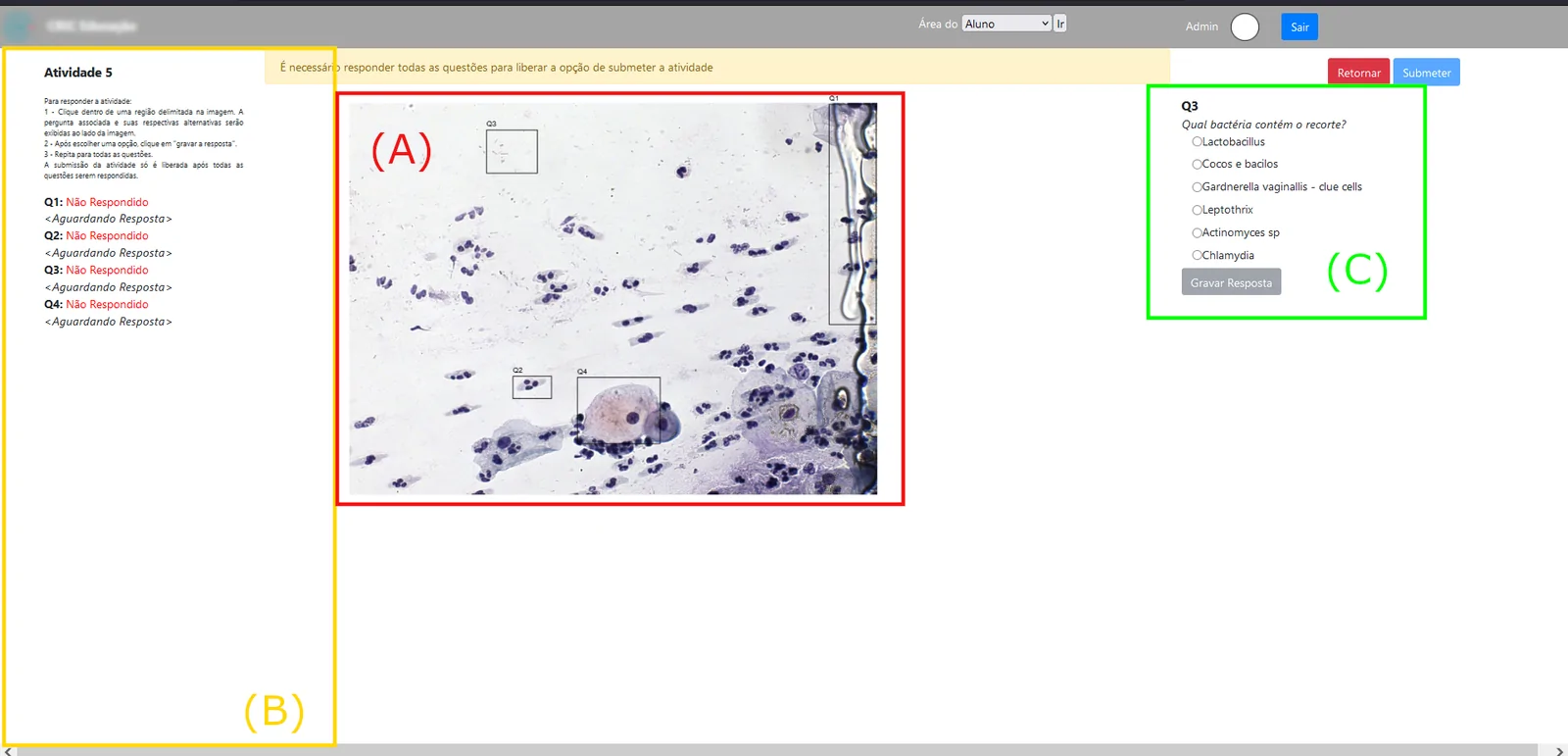
Example of the edit activity interface implemented in the system, showing how instructors create and configure learning activities for the user study.

The workflow of the content creation module follows a standardized CRUD (create, read, update, and delete) model for activities. The activity content model integrates an image with associated markings (delimited regions), which form the basis for user interaction, as illustrated in Figure 2. All images used in the activities are available in the CRIC Database [19]. Each marking represents a question in the activity and includes three elements: the coordinates for drawing the delimited region, the set of answer choices, and the correct answer. These activities can be organized thematically, allowing for structured content delivery. This format facilitates both review sessions and self-assessment. Figure 3 shows an example of the activity edit screen interface, and the system interface is in the authors’ native language (in this case, Portuguese). In region A, we have the main menu of the app for the teacher role, in which we have the options associated with the different menus of the app. In region B, we have the inputs for creating an activity, allowing us to name it, associate an image, and delimit the regions. Region C shows the image activity and the markings. In region D, we have the configuration and editing options for each of the markings created previously, allowing us to associate a question with a delimited region in the image and define the alternatives and correct answers.

##### User Interaction Module

The primary function of the user interaction module is to present the designed content to the students, permitting them to study and learn through the system. This process facilitates user interaction with the platform, generating data such as activity responses. These interactions are recorded and can be used to recommend future content tailored to the user’s needs. To achieve the system’s primary objective of supporting learning, it is essential to present the content in an interesting and nonexhaustive manner. Any challenges arising from the interaction design or content presentation format can impact the user’s success in completing the task and, over time, their overall engagement with the platform. This module manages all user interactions (regardless of their role) with the system. The framework recognizes two primary user roles: (1) student and (2) teacher. While these roles are central to the framework operation, additional roles can be implemented to address interactions beyond the main workflow.

The module implementation prioritizes basic interfaces to reduce potential challenges that a more robust interface could introduce to the interaction process. Figure 2 illustrates the activity-answering screen. In the figure, region A shows the visual component of the activity, as it was modeled in the content creation step (image+markings). In region B, instructions for using the system are presented along with a summary of the student’s responses to the activity. In region C, the question associated with the selected marking and its alternatives are presented.

However, this module is not limited to the interfaces related to the student role; it also includes all necessary interfaces to enable both roles to perform their respective actions effectively.

##### Recommendation Module

The personalization module aims to define which content should be presented to a user. Therefore, this module guides the user’s interaction with the available content in the system. This personalization involves adapting the content presentation process to the user’s characteristics and recommending content tailored to their needs and knowledge level instead of a generalized approach.

The personalization process depends on usage patterns from both the individual user and other users with similar profiles within the system. It also considers the user’s performance with the content, including their knowledge level and areas of difficulty. Consequently, the module directs recommendations to the user to address their learning needs, whether they involve acquiring new content, reinforcing existing knowledge, or consolidating understanding.

##### Interaction Between Modules

**Figure 4.**
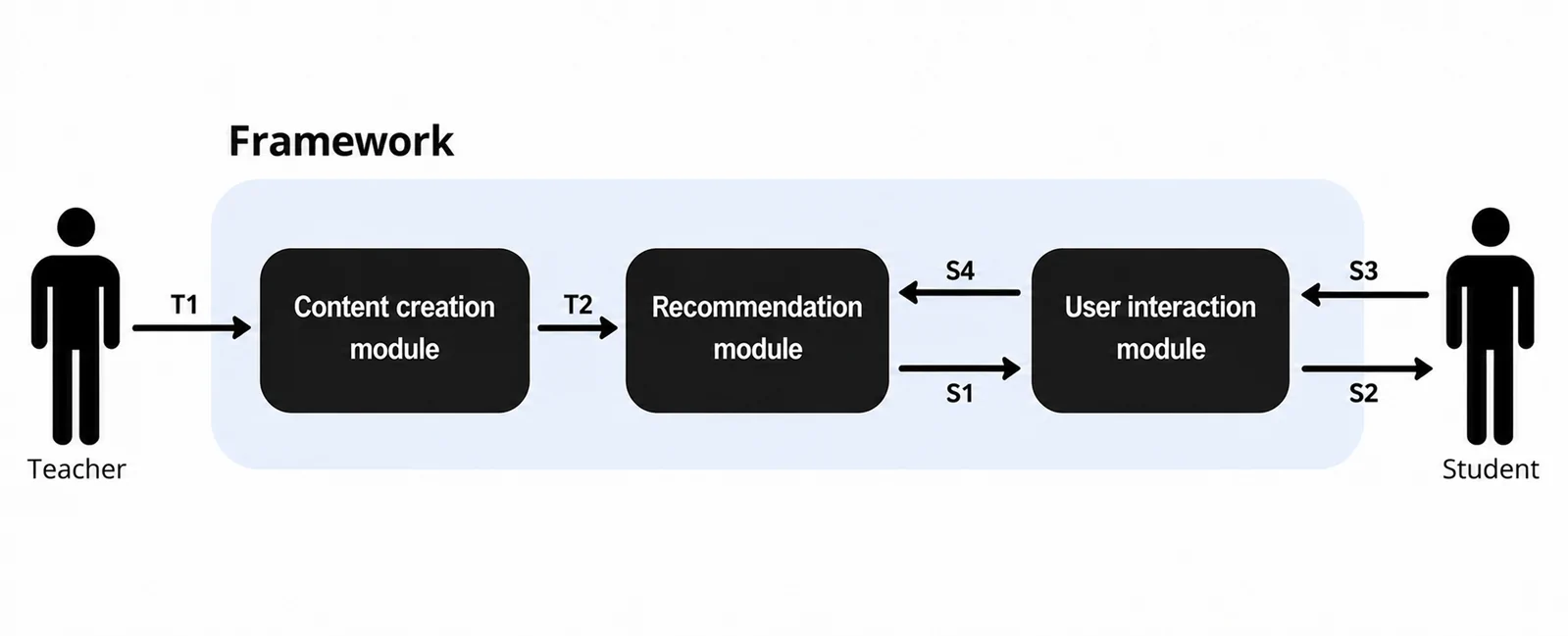
General flow of the proposed framework.

Figure 4 shows the communication between the framework modules and the users in different roles. Generally, the framework’s main workflow can be divided into two cycles. One cycle starts with the teacher creating a new piece of content that will be available in the system (T1). After that, this new content is indexed and considered to be shown to a student (T2). The other cycle starts with the system suggesting content to the student (S1). The content is presented to the student, and their interaction is collected (S2 and S3). The data collected are passed to the recommendation module to determine the following material (S4).

##### Implementation and Considerations

All framework modules have functionalities divided between the front-end and back-end of the system. Therefore, these modules were implemented for both scenarios. The front-end was developed using Angular, enabling compatibility with web browsers, while the back-end was implemented with Node.js following a REST (representational state transfer) architecture. Data storage was handled using MySQL, ensuring structured data management.

Table 1 summarizes the main differences between the framework implemented in this study and the solution described in [18]. The revised framework prioritizes improvements in the user interaction model and provides dedicated support resources for teachers.

For the purposes of this proof-of-concept test, the recommendation module is static, with an instructor-defined sequence (S1 to S2 to S3) rather than a dynamically adaptive recommendation strategy. The development and evaluation of adaptive personalization mechanisms remain subjects for future development.

**Table 1.** Comparison between systems.

Characteristic	Solution presented in a study by Keller et al [18]	This study
Modularity	No	Yes
Main interaction interface	Smartphone	Desktop
Resources for teachers	No	Yes
System adaptability	No	Yes

### Test Scenario

An evaluation was performed to verify how well the CRIC-Edu interaction model was received by users in the student role. For this purpose, an evaluation was carried out with undergraduate pharmacy students whose curriculum included cervical cytology (the base content of the system) in the course structure. Additionally, this test also evaluated the qualitative aspects of the system concerning its target audience. The evaluation scenario required each participant to interact with the system using a student-level profile and answer 24 activities (totaling 81 markings). These activities were divided into 3 subsets of content (S1, S2, and S3), aligned with the discipline’s key topics. S1 addresses the initial topics of the discipline, such as artifacts, S2 addresses content such as inflammations, and S3 addresses the final part of the discipline, focusing on lesions.

In the test scenario, participants were divided into two groups: (1) group 1, which had access to content reference material related to the set of activities, and (2) group 2, which did not have access to this resource within the system. After completing the activities, students were asked to complete a questionnaire. In addition to basic information characterizing the volunteers, such as experience in the area and use of support material, the questionnaire contained 8 statements to be evaluated on a 5-point Likert scale. These statements evaluate different aspects of the user’s interaction with the system and their ability to achieve their goal. The statements are listed below:

1. “The effort to use CRIC-Edu is low”
2. “Interactions with the CRIC-Edu system are clear and intuitive”
3. “CRIC-Edu helps you learn new information”
4. “CRIC-Edu helps clarify doubts and review content”
5. “CRIC-Edu helps improve the learning process as a whole”
6. “After using CRIC-Edu, I have more clarity about my knowledge in the area”
7. “After using CRIC-Edu, I have more confidence in carrying out the analysis task”
8. “I would use CRIC-Edu regularly to review content”

For the test, undergraduate pharmacy students enrolled in a cervical cytology course were invited to participate. The characteristics and objectives of the test were explained to each participant. Following this process, 14 volunteers were recruited. To simulate a real-world environment in which the system would be used, test participants were instructed to complete the activities at their own pace within 3 days, with a hard deadline of 15 days. After the deadline, a second open-ended questionnaire was presented to the students to collect feedback regarding possible problems they may have encountered while using the system during the test.

### Specialist Evaluation

In addition to the test with students, we also evaluated the framework and the implementation from the perspective of specialists, professionals with knowledge of the subject and experience in performing the analysis in their routine. The authors, with technical knowledge in cytology, used the system to simulate the use of both roles. For simplicity, the authors who carried out these activities will be called specialists. The specialists interacted with the system through different functionalities involving different user roles. This diversity of roles is significant because it allows for a comprehensive evaluation, since each role has access to different system functionalities. In addition to technical knowledge of cytology and different perspectives on the system, participating users also exhibited varying levels of familiarity with the system used in the test.

## Results

### User Test: Quantitative Results

The 14 volunteer students were divided equally into 2 test groups, as described previously. However, only 7 participants performed any activity, and only 4 completed all stages of the test, from answering the activities to completing the corresponding questionnaires. For the analyses presented in this study, only participants who responded to at least one activity during the test were considered for the results; thus, the data consist of the responses of 7 students, totaling 500 answered questions, an average of 71 (SD 8.34) responses out of a possible 81 per student.

For the quantitative analysis, user performance was calculated considering the correct answers for each question (marking). In this scenario, participants answered 88.36% (SD 10.30%) of all possible questions, answering correctly (on average) 69.86% (SD 14.78%). When considering all possible questions, this success rate averaged 61.73% (SD 7.87%). Table 2 shows the results divided by the test groups. In addition, the results separated by each set of activities and test group are presented in Table 3.

**Table 2.** Performance results obtained from the user test, grouped by test scenario.

Group	Time (s), mean (SD)	Answered markings (%), mean (SD)	Correct answered markings (%), mean (SD)	Correct markings (%), mean (SD)
1^a^	48.21 (19.28)	93.00 (12.12)	67.70 (20.13)	62.96 (10.97)
2^b^	35.36 (26.15)	84.88 (8.75)	71.64 (12.67)	60.80 (6.41)
All^c^	40.87 (22.65)	88.36 (10.30)	69.86 (14.78)	61.73 (7.87)

a Group 1: users with support material.

b Group 2: users without support material.

c All: all test users.

**Table 3.** Performance results obtained from the user test, grouped by activity subset and test scenario.

Subset	Group	Time (s), mean (SD)	Answered markings (%), mean (SD)	Correct answered markings (%), mean (SD)	Correct markings (%), mean (SD)
S1^a^	1^b^	54.17 (65.58)	100.00 (0.00)	70.83 (14.09)	70.83 (14.09)
S1	2^c^	32.99 (13.40)	98.44 (1.80)	69.84 (7.65)	68.75 (7.65)
S1	All^d^	42.07 (40.64)	99.11 (1.52)	70.27 (9.78)	69.64 (9.83)
S2^e^	1	52.65 (38.83)	100.00 (0.00)	84.38 (12.50)	84.38 (12.50)
S2	2	28.18 (38.25)	96.88 (4.42)	84.68 (13.77)	82.03 (11.80)
S2	All	38.67 (37.49)	98.21 (3.54)	84.55 (12.12)	83.04 (11.10)
S3^f^	1	5.38 (4.68)	66.67 (57.74)	11.76 (13.58)	7.84 (13.58)
S3	2	51.75 (98.69)	36.76 (47.64)	16.00 (8.95)	5.88 (8.32)
S3	All	31.88 (74.10)	49.58 (50.01)	13.56 (10.08)	6.72 (9.86)

a S1: artifacts.

b Group 1: users with support material.

c Group 2: users without support material.

d All: all test users.

e S2: inflammations.

f S3: injuries.

It is possible to observe that the participants’ performance metrics were impacted by their performance in the set of activities S3, especially for group 2. However, this result could be attributed to the participants’ knowledge of the content during the test, since the third set contained topics that some participants may not have been sufficiently familiar with. Another interesting aspect is that all participants performed well in the second group of activities, S2, and achieved above-average performance results in the first group. Despite group 1 having access to support material within the system, the performance difference between the 2 groups was not statistically significant.

Furthermore, the participants had an average response time of 40.87 (SD 22.65) seconds. In group 1, this value was 48.21 (SD 19.28) seconds, and in group 2, it was 35.36 (SD 26.15) seconds. These times suggest that the students performed some degree of analysis to answer the questions, indicating that their responses were not random. Furthermore, the average time of group 1 indicates that the participants may have used the reference material to answer the questions, since the average time was higher than that of group 2. However, the difference in response times between the 2 groups was not statistically significant.

### User Test: Qualitative Feedback

After completing the test activities, participants responded to an online questionnaire containing the previously described statements. However, only 4 participants completed this questionnaire. Despite the limited number of responses, participants expressed a positive perception of the system, agreeing that it was clear, intuitive, and helpful in learning new information and reviewing content. This perception is supported by their answers in positive agreement with statements such as “Interactions with the CRIC-Edu system are clear and intuitive,” “CRIC-Edu helps to learn new information,” “CRIC-Edu helps to clarify doubts and review content,” and “CRIC-Edu helps to improve the learning process as a whole.”

However, participants also reported some difficulties with the system, as evidenced by their responses in disagreement with the statements “The effort to use CRIC-Edu is low” and “After using CRIC-Edu, I have more clarity about my knowledge in the area.” These negative impressions and reported difficulties with the system may be caused by the initial learning curve required to understand and properly use the system. While the interaction format is designed to be simple and similar to laboratory tasks, participants need time to familiarize themselves, which could impact their initial experience.

### Comparison Between Approaches

The activities utilized in the user test were also presented in [18], enabling a direct comparison of accuracy results. To ensure consistency, the results presented in [18] were filtered to consider only the 81 markings used in our test scenario. However, it is important to highlight that the evaluation performed in [18] did not collect response times, so this comparison is impossible. Additionally, it is also impossible to compare some subjective aspects observed, given the difference in the system used for the test.

Tables 4 and 5 summarize the results from the SAP and RAP test scenarios, providing a baseline for evaluating the metrics observed in this study.

It is noteworthy that although the average responses obtained are similar to the scenarios described, the SAP and RAP scenarios achieved a higher average of correct answers. It is important to highlight that in the SAP and RAP test scenarios, the participants performed the test as a parallel activity to the course, and the division of the activities was different in each scenario. Furthermore, the test structure permitted the content covered in the activities to already be clearer for some participants, as discussed in [18].

**Table 4.** Average performance results from the preliminary approach [18], limited to the test activities used in this study.

Group	Answered markings (%), mean (SD)	Correct answered markings (%), mean (SD)	Correct markings (%), mean (SD)
SAP^a^	89.88 (7.46)	85.80 (9.23)	77.11 (10.43)
RAP^b^	87.52 (7.00)	83.23 (8.65)	72.84 (9.58)

a SAP: short academic period.

b RAP: regular academic period.

**Table 5.** Average performance results from the preliminary approach [18], grouped by activity subset and user group.

Subset	Group	Answered markings (%), mean (SD)	Correct answered markings (%), mean (SD)	Correct markings (%), mean (SD)
S1^a^	SAP^b^	98.56 (8.91)	81.93 (11.43)	80.75 (13.60)
S1	RAP^c^	99.31 (2.08)	78.32 (9.20)	77.78 (8.76)
S2^d^	SAP	86.56 (3.90)	91.99 (12.09)	79.63 (10.81)
S2	RAP	87.50 (0.00)	90.08 (7.09)	78.82 (6.21)
S3^e^	SAP	79.76 (12.92)	82.15 (16.24)	65.53 (14.46)
S3	RAP	65.36 (33.85)	80.00 (30.68)	52.29 (28.28)

a S1: artifacts.

b SAP: short academic period.

c RAP: regular academic period.

d S2: inflammations.

e S3: injuries.

Except for the SAP scenario, the answers data for all observed groups were relatively small. Thus, to assess whether these data exhibited statistical similarity, they were evaluated with the Shapiro-Wilk test, as shown in Table 6. Results smaller than 10e-10 were represented as <.001. The analysis indicates that most answer datasets did not follow a normal distribution at a significance level of α=.05. However, for the correct-answer data, some samples did present a normal distribution, as highlighted in Table 7 for α=.05.

Given these data characteristics, a Kruskal-Wallis test was performed to compare the 3 scenarios (SAP, RAP, and this study) to evaluate the statistical similarity of these data. The results are shown in Table 7; results smaller than 10e-10 were represented as <.001. The analysis indicates that the answer data from this test scenario are statistically similar to those observed in the SAP and RAP scenarios. However, the same does not occur for the correct answers. These findings suggest that the test participants had a similar engagement in using the system and responding to the activities, but obtained different performance levels, probably resulting from differences in their knowledge about the content during the test.

Considering the impact of S3 on the test results, the analyses described above were also performed for the combination of S1 and S2, as shown in Table 6. In all cases, the observed data demonstrate a consistent pattern, aligning with established benchmarks in perceptual learning. This indicates that the answers for S3 do not impact the analysis of the overall data.

**Table 6.** Results of the Shapiro-Wilk normality test applied to all data groups (α=.05).

Group	Subset	*P* value (answers)	*P* value (correct answers)
This study	All	.02	.41
This study	S1^a^	<.001	.80
This study	S2^b^	<.001	.71
This study	S3^c^	.02	.01
This study	S1 and S2	<.001	.87
SAP^d^	All	<.001	<.001
SAP	S1	<.001	.002
SAP	S2	<.001	<.001
SAP	S3	<.001	<.001
SAP	S1 and S2	<.001	<.001
RAP^e^	All	<.001	.03
RAP	S1	<.001	.19
RAP	S2	—^f^	.02
RAP	S3	<.001	.03
RAP	S1 and S2	<.001	.01

a S1: artifacts.

b S2: inflammations.

c S3: injuries.

d SAP: short academic period.

e RAP: regular academic period.

f Not applicable.

**Table 7.** Results of the Kruskal-Wallis statistical test evaluating difference across activity subsets × user responses (α=.05).

Subset	Answers		Correct answers	
	*P* value	Conclusion	*P* value	Conclusion
All	.28	Similar	<.001	Not similar
S1	.09	Similar	.04	Not similar
S2	<.001	Not similar	.25	Similar
S3	.41	Similar	<.001	Not similar
S1 and S2	.03	Not similar	.14	Similar

### Specialist Evaluation

The specialists’ perception of the system was positive, reporting a good experience using it. However, when evaluating the system from each user role, the perception from the student’s side was very positive, which aligns with the feedback collected from the students in the test. On the teacher’s side, it was positive but with limitations. These conclusions are directly correlated with some of the statements made by specialists during the interview phase. In the interview, one of the specialists stated regarding the tool: “I found it very useful, very interesting,” while another stated “The tool is well defined, what needs to be answered is very clear” and “The markings clearly indicating what I have to answer are very clear, as is the number of questions and options to choose from.” While in the teacher role, some of the difficulties were reported: “As a teacher, I had difficulties putting together these activities, perhaps because I didn't understand the system part.”

These difficulties can be caused by the system’s learning curve, that is, understanding the terminology and navigating where to perform the different tasks in the role of teacher. The positive perception of the specialists in the student role, combined with the test participants’ feedback, reinforces that the objective of delivering a simple interaction model and reducing the impact of this interaction on the learning task was achieved.

In general, the specialists agreed that the system fulfills its objective of serving as a tool to support the learning process. Statements such as: “As you mark the answers, you’ll know if they’re right or wrong, which helps you review why you’re making the mistake” show the specialists’ perception. This perception is primarily derived from the system’s interaction model, which presents activities that simulate the view of an optical microscope, with highlighted areas. The specialists evaluated this interaction format as an excellent way to exemplify theoretical knowledge in classes and expose students to specific and unusual situations, in addition to providing support material for the class’s development, as described by a specialist: “I'll have to delimited the area, present the situation, but in return I'll have that clearly defined and saved, for example, to compare later.” Also, they appreciated that this interaction can be accessed via desktop and the internet and is not limited to a laboratory with specific equipment. In addition to the terminology used in the system, the specialists did not highlight any problems or difficulties with usability. However, this challenge with the system’s terminology is also associated with the initial learning curve required to use the system. It is enhanced by potential ambiguities in the system’s native language (Portuguese).

Finally, the specialists evaluated the system’s inclusion in their routine as an advantage. However, it is necessary to assess whether this positive reception is maintained with continued use. This requires a more extended test with broad participation.

## Discussion

### Main Findings

This study introduced a computational framework designed to enhance perception skills in cervical cytology through interactive and adaptive visual exercises, an alternative model of interaction between the professional and the samples. According to the objectives presented in the Introduction, the proposed framework successfully maintained student performance while improving usability, interaction quality, and overall user experience compared with previous approaches. These findings suggest that the proposed framework can support cytology education without compromising learning effectiveness while offering a more flexible, scalable, and specialist-oriented interaction model.

The framework also demonstrated the potential to organize educational activities as structured computational objects that can record perceptual interactions and support future personalization strategies. In addition, the results indicate that exposing students to both common and rare clinical scenarios may help bridge the gap between theoretical knowledge and practical expertise in cervical cytology.

### Interpretation of Findings, Educational Implications, and Comparison With Previous Works

Compared with the previous approach proposed by Keller et al [18], the present framework introduces several architectural and educational improvements. The modular architecture allows the inclusion of new interaction models, features, and functionalities into the overall interaction flow without compromising the system’s stability. Such flexibility expands the platform’s future adaptability and facilitates its evolution in line with educational demands.

Another benefit of the proposed framework concerns the refined or newly created graphical user interfaces capable of delivering the proposed interaction model. In a study by Keller et al [18], there were no user interfaces that allowed education professionals to monitor students’ interactions. In contrast, the proposed framework presents this information through a user interface that supports the course’s execution.

Specialist feedback also highlighted the importance of interaction mechanisms that support answer clarification and collaborative discussion. Since cytology analysis is not an exact science, different interpretations may occur and must be clarified. This demand for interaction can be divided into two types of interaction: (1) answer clarification, which involves explaining why a specific region is identified as option A rather than option B, and (2) assistance with complex analysis, which focuses on helping users clarify a doubt they had in their activities. These observations align with discussions presented in a study by Keller et al [18,20], which emphasized the importance of collaborative support systems in specialized visual analysis environments.

The proposed framework is also aligned with principles of perceptual learning in medical education, in which expertise is progressively developed through repeated exposure to structured visual patterns and immediate interpretive feedback [14,15]. These observations are consistent with prior studies on perceptual learning in medical education, which demonstrated that repeated visual exposure and feedback-based interaction improve diagnostic expertise [14,15,17].

In cytology, diagnostic proficiency depends heavily on the ability to recognize subtle morphological variations across a large number of cases. By exposing students to both common and rare lesion patterns in an interactive environment, the framework supports the gradual refinement of visual discrimination skills and diagnostic reasoning.

The experimental protocol adopted in this study aimed to simulate a realistic educational scenario in which students could freely interact with the platform without rigid usage constraints. The results also suggest that incomplete participation was more closely associated with prioritization and engagement factors than with technical difficulties or usability barriers. Since communication channels were available and no relevant system-related problems were reported, it is possible to infer that noncompletion behavior reflects the voluntary nature of the activity rather than limitations of the framework itself. Similar behavior patterns were also observed in the RAP environment [18], where optional educational activities tended to receive lower prioritization when not directly associated with formal course evaluation.

In the context of specialist training, the framework also demonstrated potential to support continuing education processes. Cytology professionals frequently require ongoing updates through review courses and professional events, particularly given the complexity and variability of visual interpretation tasks. In this scenario, the proposed framework may support both structured educational methodologies and continuous professional development activities.

This process enables quantification of individual performance and identification of knowledge gaps. Based on these insights, professionals can tailor their training strategies or have the system support them in following a personalized learning path based on their data (response times, accuracy, and answer patterns). As a result, the platform serves as a diagnostic tool to identify individual difficulties and propose adapted study plans, increasing the overall effectiveness of cytology teaching and professional development [3,15]. This may improve the effectiveness of cytology training and ongoing professional development, particularly compared with traditional, less adaptive training approaches.

Another important aspect concerns the framework’s potential to support adaptive learning strategies. By recording user interactions, response times, and answer patterns, the system creates opportunities for future personalization mechanisms that can adapt educational pathways to individual performance and learning difficulties. Such approaches are consistent with adaptive education models that seek to optimize learning efficiency by tailoring content and difficulty levels to the learner’s evolving expertise.

Although the framework was developed and evaluated in the context of cervical cytology, its modular architecture was designed to facilitate adaptation to other domains that depend on visual pattern recognition, including pathology, histology, radiology, and digital pathology.

This adaptability is supported by the separation between data, interaction, and processing modules, which allows domain transfer with minimal system-level modifications. In practice, adaptation can occur in two different ways: (1) replacing the content module data, that is, changing the data without system modifications; or (2) extending the user interface module to offer the specific interaction models, combined with the inclusion of the new content data in the system database.

These characteristics suggest that the framework may serve as a scalable computational environment for perceptual learning in health care education. Beyond cervical cytology, the proposed model contributes to the broader discussion of computational support for the development of visual expertise in medical training. By integrating structured interaction, explainable educational workflows, and behavioral data collection, the framework creates opportunities for scalable and adaptive learning environments in domains where visual interpretation skills are critical.

The CRIC-Edu framework has the potential to significantly influence the training of cytology professionals by fostering decision-making skills in complex clinical environments. Integrating simulated laboratory experiences into the framework allows students to repeatedly practice visual diagnostic tasks, strengthening critical analytical and reasoning skills. This is consistent with prior studies highlighting digital and simulation-based resources in pathology and medical education [3,9,12,13]. This iterative exposure improves their ability to recognize patterns and enhances their expertise in evaluating cellular lesions.

In addition, the platform provides professionals with access to a broader range of cases and scenarios than is possible through traditional laboratories or textbooks. By exposing students to structured, diverse, and rare cases, the framework helps build mental models that health professionals rely on to make rapid and accurate decisions in complex environments. It enables students to refine their interpretive abilities and develop clinical intuition, a skill for identifying and prioritizing critical issues under pressure.

The framework also enables students to engage with advanced and rare scenarios that are not always accessible in traditional training programs. By simulating these high-complexity situations, the system encourages learners to develop confidence and competence in tackling ambiguous or challenging cases.

Integrating real-time feedback and reference materials within the platform promotes evidence-based, feedback-guided learning, a principle also emphasized in adaptive learning and the development of perceptual expertise [14,15]. As students receive immediate insights into their performance, they are encouraged to reflect on their diagnostic choices, fostering a habit of seeking evidence to inform clinical decisions. This approach reduces anxiety and improves performance in time-sensitive or high-stakes tasks, equipping students to excel under pressure.

Additionally, the system’s scalability and adaptability turn it into a valuable tool for training health professionals globally, addressing disparities in educational resources and ensuring equitable access to high-quality training.

### Limitations

The main limitation of this study concerns the reduced number of participants who completed all stages of the evaluation protocol. For the quantitative analysis, responses from 7 distinct users were collected, totaling 500 answered questions, while the qualitative analysis involved 4 participants. Although these data were sufficient to identify preliminary behavioral patterns and interaction tendencies, the limited sample size restricts the generalizability of the findings.

Additionally, the statistical comparisons presented in this study should be interpreted as exploratory and descriptive rather than confirmatory. A larger and more diverse participant population is necessary to validate the observed trends and strengthen the robustness of the conclusions.

The anomaly observed in the S3 subset also suggests that certain complex diagnostic categories may require additional refinement in the adaptive interaction mechanisms of the framework to better support visual differentiation and ambiguity resolution. Furthermore, because participation was voluntary, motivational and prioritization factors may have influenced user engagement and task completion rates.

Another limitation concerns the absence of longitudinal evaluation. This study focused on immediate interaction and performance indicators, preventing conclusions regarding long-term retention of perceptual skills acquired through the platform. Future studies should investigate whether repeated interactions with the framework contribute to sustained improvements in diagnostic expertise.

### Conclusion

This study proposed and evaluated the CRIC-Edu framework, a computational environment designed to support perceptual learning in cervical cytology through structured visual inspection activities, interaction traceability, and behavioral data collection. The results demonstrated that the framework maintained learning performance while improving usability, interaction quality, and overall user experience compared with previous approaches. These findings indicate that the proposed approach can effectively support cytology education without compromising learning outcomes while providing a more flexible and scalable educational environment.

Beyond its application to cervical cytology education, this study contributes to the broader field of computational support for perceptual learning in health care education. By combining explainable educational workflows, structured interaction models, behavioral data analysis, and adaptive learning principles, the framework illustrates how a digital educational environment can support the progressive development of visual expertise. Such characteristics are particularly relevant in health care disciplines where diagnostic competence relies heavily on repeated visual interpretation, guided practice, and continuous professional development.

The proposed framework also demonstrates the potential of modular educational platforms to extend to other domains, such as pathology, histology, radiology, and digital pathology, enabling the development of scalable and remotely accessible educational environments. More broadly, this study reinforces the importance of integrating computational technologies with evidence-based educational principles to expand access to specialized training, support lifelong learning, and reduce disparities in professional qualification across different health care settings.

Future work will focus on longitudinal evaluation of perceptual skill retention in a larger population, expansion of the image database, and investigation of adaptive learning mechanisms that generate personalized educational pathways based on user interaction patterns and performance data. These developments may further strengthen the role of computational educational technologies as promising tools for expanding access to high-quality training in visually intensive and personalized health care domains.

## Abbreviations

CRUD: create, remove, update, and delete
RAP: regular academic period
REST: representational state transfer
SAP: short academic period

## References

[1] Guze PA (2015). Using technology to meet the challenges of medical education. Trans Am Clin Climatol Assoc.

[2] Aziz Hussin A (2018). Education 4.0 made simple: ideas for teaching. Int J Educ Lit Stud.

[3] Dankbaar MEW, de Jong PGM (2014). Technology for learning: how it has changed education. Perspect Med Educ.

[4] Dargham J, Saeed D, Mcheick H (2012). E-learning at school level: challenges and benefits.

[5] Thakkar SR, Joshi HD (2015). E-learning systems: a review.

[6] Maley MAL, Harvey JR, Boer W de, Scott NW, Arena GE (2008). Addressing current problems in teaching pathology to medical students: blended learning. Med Teach.

[7] Wiecha J, Heyden R, Sternthal E, Merialdi M (2010). Learning in a virtual world: experience with using second life for medical education. J Med Internet Res.

[8] Souto Bahia N, da Silva WR, Bacchi Vianna J, Gabriel Rodrigues H, Brazão Silva MT, Bacchi RR (2019). O uso das TDIC’s como estratégia para aprendizagem em morfologia microscópica [Article in Portuguese]. Informática na educação: teoria & prática.

[9] Guiter GE, Sapia S, Wright AI, Hutchins GGA, Arayssi T (2021). Development of a remote online collaborative medical school pathology curriculum with clinical correlations, across several international sites, through the Covid-19 pandemic. Med Sci Educ.

[10] Hanna MG, Reuter VE, Ardon O (2020). Validation of a digital pathology system including remote review during the COVID-19 pandemic. Mod Pathol.

[11] Melo CA, Sousa MS (2021). Tecnologia educacional como estratégia integrativa de complementação na formação de estudantes e profissionais da área da saúde: Revisão integrativa [Article in Portuguese]. Res Soc Dev.

[12] Chen LS, Lay YJ, Yang CC, Chang SH (2015). A virtual microscope system for histology learning in education. 8th International Conference on Ubi-Media Computing.

[13] Manou E, Lazari EC, Lazaris AC, Agrogiannis G, Kavantzas NG, Thomopoulou GE (2022). Evaluating e-learning in the pathology course during the COVID-19 pandemic. Adv Med Educ Pract.

[14] Sasaki Y, Nanez JE, Watanabe T (2010). Advances in visual perceptual learning and plasticity. Nat Rev Neurosci.

[15] Kellman PJ, Krasne S (2018). Accelerating expertise: perceptual and adaptive learning technology in medical learning. Med Teach.

[16] Bütün Ayhan A, Aki E, Mutlu B, Aral N (2015). A study of conceptual development and visual perception in six-year-old children. Percept Mot Skills.

[17] Marris J, Perfors A, Mitchell D How effective is perceptual training? Evaluating two perceptual training methods on a difficult visual categorisation task. https://escholarship.org/uc/item/0wm2w81m.

[18] Keller BNS, Rezende MT, Machado TM, Delabrida SE, Carneiro CM, Bianchi AGC (2022). Perceptions on the use of an online tool in the teaching-learning process in microscopy. Int Conf Enterp Inf Syst.

[19] Rezende MT, Silva R, Bernardo FO (2021). Cric searchable image database as a public platform for conventional pap smear cytology data. Sci Data.

[20] Keller B, Guimarães TD, Malaquias P (2021). CitoFocus: uma plataforma para colaboração e aprendizado em citopatologia [Article in Portuguese]. Simpósio Brasileiro de Computação Aplicada à Saúde.

